# Cross-modal impact of auditory experience on visual cortical entrainment in children who are hard-of-hearing

**DOI:** 10.1162/IMAG.a.1001

**Published:** 2025-11-10

**Authors:** Elizabeth Heinrichs-Graham, Zhiying Shen, Wai Hon Lee, Amanda A. Benavente, Jacob A. Eastman, Michaela R. Frenzel, Alex I. Wiesman, Tony W. Wilson, Elizabeth A. Walker, Ryan W. McCreery

**Affiliations:** Cognitive and Sensory Imaging Laboratory, Institute for Human Neuroscience, Boys Town National Research Hospital, Omaha, NE, United States; Center for Pediatric Brain Health, Institute for Human Neuroscience, Boys Town National Research Hospital, Omaha, NE, United States; Department of Pharmacology and Neuroscience, Creighton University, Omaha, NE, United States; Dynamic Imaging of Cognition and Neuromodulation Laboratory, Institute for Human Neuroscience, Boys Town National Research Hospital, Omaha, NE, United States; Department of Biomedical Physiology and Kinesiology, Simon Fraser University, Burnaby, Canada; Wendell Johnson Speech and Hearing Center, Department of Communication Sciences and Disorders, University of Iowa, Iowa City, IA, United States; Audibility, Perception, and Cognition Laboratory, Boys Town National Research Hospital, Omaha, NE, United States

**Keywords:** hearing loss, oscillations, visual steady state, sensory experience, developmental neuroimaging, hearing aids

## Abstract

Cross-modal neuroplasticity is a well-studied phenomenon whereby in the absence of sensory input (e.g., in individuals who are deaf or blind), the sensory cortices of the affected modality are “taken over” by the unaffected sensory systems. This neuroplasticity is often coupled with changes in the dynamics of the unaffected primary sensory cortices themselves. Nonetheless, whether these cross-modal changes extend to those with mild-to-severe sensory loss where sensory input is degraded but not absent, as is the case for children who are hard-of-hearing (CHH), remains poorly understood. Visual entrainment, sometimes referred to as the visual steady-state response, is the process by which the primary visual cortices entrain to the frequency of a rhythmic exogenous visual stimulus. Importantly, visual entrainment dynamics are thought to serve as an important proxy for how visual stimuli are processed in the brain. The current study sought to identify the impact of mild-to-severe hearing loss and individual differences in auditory experience on visual entrainment dynamics in youth. To this end, CHH and an age- and sex-matched group of children with normal hearing (CNH) were presented with a visual entrainment stimulus that flickered at 15 Hz during magnetoencephalography. Neural responses to the fundamental (15 Hz) and first harmonic (30 Hz) were imaged using beamforming, and the power envelope of the peak responses was extracted as a function of time and submitted to linear mixed effects modeling. We found a significant group-by-time interaction, whereby CHH exhibited a stronger increase in power during entrainment than CNH, suggesting altered entrainment dynamics. We also ran whole-brain correlations between 15 and 30 Hz neural responses and hearing aid use in CHH, controlling for degree of hearing loss. We found a significant negative relationship between hearing aid use and activity in the left primary auditory cortex and left lateral parietal cortex, such that CHH who consistently wore their hearing aids showed the smallest amount of cross-modal neuroplasticity in these regions. Crucially, reductions in cross-modal neuroplasticity in the left lateral parietal cortex were related to better verbal outcomes. These data provide important new information regarding how consistent auditory experience may serve to normalize the neural dynamics serving sensory processing, and how these changes may cascade into improvements in verbal ability in CHH.

## 1 Introduction

Children with mild-to-severe hearing loss (i.e., children who are hard-of-hearing [CHH]) are at a heightened risk of delays in speech, language, and academic outcomes compared with children with normal hearing (CNH), despite early detection and intervention with hearing aids (Emmett & Francis, 2014; Khairi Md Daud et al., 2010; McCreery & Walker, 2022; McCreery et al., 2017, 2019; Moeller, 2007; Moeller & Tomblin, 2015; Tomblin, Oleson, Ambrose, Walker, & Moeller, 2020; Tomblin et al., 2015; Walker et al., 2020; Werfel et al., 2020). Moreover, CHH exhibit immense variability in cognitive and language outcomes such that some are able to catch up with their normal hearing peers, whereas others fall persistently behind (McCreery et al., 2017; Tomblin, Oleson, Ambrose, Walker, McCreery, et al., 2020). Recent research has sought to determine the mechanisms that underlie this variability in language and cognitive ability, and there is accumulating evidence that the quality and quantity of auditory experience, including how often children wear their hearing aids and how well their hearing aids restore access to speech sounds, play a significant role (Farquharson et al., 2022; Heinrichs-Graham, Walker, Eastman, et al., 2022; Heinrichs-Graham, Walker, Taylor, et al., 2022; McCreery & Walker, 2022; Tomblin, Oleson, Ambrose, Walker, McCreery, et al., 2020; Tomblin et al., 2015; Walker, Holte, et al., 2015; Walker et al., 2020; Wiseman et al., 2023). From a more basic sense, variability in auditory experience, due to hearing loss itself and/or variability in hearing aid intervention, inherently results in alterations in the auditory signals that reach the brain (Kitzes & Semple, 1985; Kral et al., 2001; Zhang et al., 2002), and these differences in cortical auditory input likely have cascading effects on other sensory and cognitive processes (Kral & Sharma, 2012). Thus, a fundamental understanding of how differences in auditory experience are related to alterations in sensory processing may be an important first step in understanding how these mechanisms lead to differences in language and cognitive processing.

Cross-modal neuroplasticity is a widely accepted phenomenon in cases of complete sensory loss, whereby the sensory cortices of the affected modality are “taken over” (i.e., recruited) by another sensory modality, and this is sometimes coupled with changes in the physiology of the sensory cortices primarily responsible for processing the intact senses; excellent reviews include (Kral & Sharma, 2023; H.-K. Lee & Whitt, 2015; Merabet & Pascual-Leone, 2010). Cross-modal neuroplasticity is supported by a myriad of both animal and human studies. For example, in humans, studies have consistently found cross-modal neural reorganization; for example, individuals who are deaf consistently exhibit activation in the auditory cortex in response to visual (Alencar et al., 2019; Bottari et al., 2010; Finney et al., 2003; Gordon et al., 2011; Retter et al., 2019; Sharma et al., 2015) and somatosensory stimuli (Auer et al., 2007; Hennesy et al., 2022), while auditory tasks consistently elicit activity in the occipital cortex in individuals who are blind (Hötting & Röder, 2009; Kujala et al., 2000; Sabourin et al., 2022). Few studies have looked at the spatiotemporal neural oscillatory dynamics of sensory processing in individuals who are deaf or blind, but these have shown significant elevations in frequency-specific oscillatory amplitude in the deprived sensory area during processing of other sensory modalities (Bednaya et al., 2021; Schepers et al., 2012). Functionally, *increased* cross-modal neuroplasticity in response to visual stimuli in deaf individuals is often coupled with an enhanced ability to process visual information, including visual target detection (especially in the periphery; Alencar et al., 2019; Bednaya et al., 2021; K. R. Lee et al., 2022) and visual motion processing (Bosworth & Dobkins, 2002; Retter et al., 2019; see Alencar et al., 2019 for a review). However, recent work suggests that in both children and adults with severe-to-profound hearing loss, there are *decreases* in cross-modal plasticity following cochlear implantation (Kral & Sharma, 2023; Sharma & Glick, 2016; Sharma et al., 2015), and the amount of decrease in cross-modal neural representation following cochlear implantation is related to improved speech outcomes (Doucet et al., 2006; Paul et al., 2024). Taken together, this literature suggests that the impact of cross-modal neuroplasticity on visual processing outcomes seems to be dynamic and sensitive to auditory experience. There is also limited work suggesting that CHH exhibit enhancements in visual attention processing, though the literature is mixed and most studies combined CHH and children with severe-to-profound hearing loss (Bell et al., 2020; Jerger et al., 2020; Tharpe et al., 2008). Nonetheless, it is clear that the brain’s ability to adapt to differences in sensory experience substantially impacts behavior in a dynamic manner, both in the unaffected and affected sensory domains.

Unfortunately, much less is known about the underlying neural dynamics of sensory processing when a sensory modality is degraded but not completely absent, as in the case of CHH, who often have significant residual auditory function or access that is made at least somewhat more available with hearing aids. One study showed that adults with asymmetric hearing loss exhibited a nonlinear increase in cortical responsivity in the auditory cortices during sound processing relative to objective loudness level (Van Eeckhoutte, Spirrov, et al., 2018), in contrast to adults with normal hearing (Van Eeckhoutte, Wouters, et al., 2018). Moreover, recent work from our laboratory found significant elevations in stimulus-related somatosensory activity in CHH relative to CNH, suggesting that CHH exhibit compensatory increases in neural activity within primary sensory networks (Heinrichs-Graham et al., 2023). Moreover, CHH who were best able to account for this compensatory increase in somatosensory responsivity also had the best verbal and academic outcomes (Heinrichs-Graham et al., 2023). These data suggest, at least preliminarily, that individuals with diminished residual hearing show a nonlinear relationship between stimulus intensity and cortical sensory responsivity in a given primary sensory cortex, and that the optimization of these sensory dynamics has some behavioral relevance. However, it still remains to be seen whether individual differences in auditory experience with CHH are related to altered sensory dynamics in the visual domain, or whether CHH also exhibit cross-modal neuroplasticity within primary auditory cortices in response to extramodal sensory input. More broadly, there is also limited work investigating how cross-modal neuroplasticity is reflected in areas that are critical to multisensory integration, including the lateral prefrontal, superior temporal, and lateral parietal cortices. For example, the lateral posterior parietal cortex has been reliably associated with multimodal integration (Bolognini et al., 2009; Limanowski & Blankenburg, 2018; Regenbogen et al., 2018; Sereno & Huang, 2014; Ursino et al., 2014), in addition to other cognitive and integrative functions (Bruner et al., 2023; Corbetta & Shulman, 2011; Hickok & Poeppel, 2004; Humphreys et al., 2022; Kemmerer et al., 2022; Poeppel, 2014). It is possible that one or more of these areas would be differentially impacted by changes in sensory experience and subsequent alterations in sensory processing within the primary sensory cortices, but this has not been probed to date.

Sensory entrainment provides a rich, multifaceted framework with which to study sensory processing. Sensory entrainment, also called a sensory steady-state response or frequency-following response, is the process by which populations of neurons synchronize their activity to an exogenous rhythmic stimulus (Lakatos et al., 2019). A wealth of literature has established that primary sensory systems entrain to rhythmic auditory, visual, and somatosensory stimulation (Albouy et al., 2022; Herrmann, 2001; Lakatos et al., 2019; Regan, 1966). Of particular relevance, the visual steady-state response (VSSR), or visual entrainment response, is characterized by an oscillatory entrainment response at both the fundamental frequency of stimulation and its harmonics, and lasts from ~100 ms after the onset of the stimulus until ~200 ms after stimulus offset. Broadly speaking, sensory entrainment is thought to serve as a robust measure of the underlying functional properties of a given sensory system (i.e., the system’s integrity), and various neurophysiological metrics, including amplitude, latency (i.e., the amount of time between the onset of an exogenous stimulus and the onset of entrainment, or the amount of time between the termination of an exogenous stimulus and the offset of the neural entrainment response), and slope (i.e., how fast the system entrains and how stable the entrainment response is once established) have been quantified with relation to cognitive measures in normative and clinical populations (Gallina et al., 2023; Springer et al., 2022, 2023; Wiesman & Wilson, 2019; Wiesman et al., 2019). Nonetheless, to date, the impact of mild-to-severe hearing loss on visual entrainment dynamics has not been characterized, and an investigation of these dynamics in CHH may help determine how variability in auditory experience (e.g., degree of hearing loss, amount of hearing aid use) affects sensory processing in the context of cross-modal neuroplasticity.

The current study aims to characterize the impact of mild-to-severe hearing loss on the neural dynamics underlying visual entrainment, as well as to determine whether variability in auditory experience relates to alterations in visual entrainment processing. To this end, we enrolled a group of CHH and CNH of ages 7–15 years old to complete a passive viewing of a centrally presented white dot that flickered at 15 Hz during magnetoencephalography (MEG). We hypothesized that CHH would exhibit altered visual entrainment dynamics within the occipital cortices, indicative of a reduced stability of entrainment. We also hypothesized that auditory experience would modulate these dynamics, such that those with the most auditory experience would exhibit dynamics that were most similar to CNH peers. Finally, we hypothesized that individuals with the least cumulative auditory experience would show elevated cross-modal neuroplasticity in the primary auditory cortices, in line with research in those with severe-to-profound hearing loss.

## 2 Methods

### 2.1 Participant recruitment

A total of 42 participants aged between 7 and 15 years (25 CNH and 17 CHH) were recruited and consented to participate in the study. Potential participants were excluded due to any of the following: a history of any central nervous system illness, current major neuropsychiatric disorder, history of head trauma (parent-reported concussion or traumatic brain injury, loss of consciousness due to a blow to the head, etc.), current substance use, and/or irremovable ferromagnetic material in or on the body (e.g., dental braces or bridges, battery-operated implants). After a complete description of the study was given to participants, written informed consent was obtained from the parent/guardian of the participant and informed assent was obtained from the participant following the guidelines of the local institutional review board (IRB # 689-19-EP), which approved the protocol. Researchers were blinded to group membership during all preprocessing and processing of MEG and behavioral data until statistically necessary to avoid any potential bias.

### 2.2 Neuropsychological testing and questionnaires

All participants completed a battery of neuropsychological tests, including the Wechsler Abbreviated Scale of Intelligence (WASI-II; McCrimmon & Smith, 2013) and the category fluency and letter fluency sections of the verbal fluency subtest on the Delis–Kaplan Executive Function System (D-KEFS; Homack et al., 2005) to characterize various aspects verbal and nonverbal cognitive function. Briefly, the WASI-II consists of four subtests: the Vocabulary and Similarities subtests can be combined to create a Verbal Comprehension Index (VCI), which is a measure of verbal ability, while the other two subtests, Block Design and Matrix Reasoning, can be combined to create the Perceptual Reasoning Index (PRI), which is a measure of nonverbal ability. All four subtests are combined to create an overall intelligence quotient score (FSIQ-4), which is reflective of general cognitive ability. Parents also completed the Edinburgh Handedness Survey (Oldfield, 1971) to determine handedness, as well as the Vanderbilt ADHD Diagnostic Parent Rating Scale (Anderson et al., 2022; Bard et al., 2013) to characterize ADHD symptomatology. The parents of two CNH and one CHH did not complete the Vanderbilt ADHD Diagnostic Parent Rating Scale. Finally, parents or guardians filled out questionnaires regarding their child’s hearing aid use during the school year, summer, and weekends (e.g., “During the school year, how many hours a day does your child wear the aids Monday–Friday? Saturday–Sunday?”). We then calculated the total hours of hearing aid use per week, Monday through Sunday. For all analyses, we used the average number of hours/week participants wore their hearing aids during the school year, including weekends (Monday–Sunday).

### 2.3 Audiometric testing

We characterized degree of hearing loss by calculating their better-ear pure-tone average (BEPTA) for all CHH using their most recent clinical audiogram, which was completed within 1 year of the test visit and provided with parent consent. Audiograms consisted of air-conduction audiometric thresholds measured at octave frequencies from 500 to 4000 Hz. Pure-tone averages (PTA) were first calculated by averaging the threshold values from 500, 1000, 2000, and 4000 Hz for each ear; the lower PTA between each participant’s ears (i.e., BEPTA) was used to represent degree of hearing loss in all statistical analyses. CNH were screened at octave frequencies from 500 to 4000 Hz to determine that their hearing thresholds were within normal ranges (i.e., lower than 20 dB).

### 2.4 Experimental paradigm

Prior to MEG recording, participants were seated in a nonmagnetic chair with their head positioned within the MEG array. Participants were instructed to remain still and fixate on a small white circle that flickered at 15 Hz (i.e., presentation every 66.67 ms) for 1.0 s, with a jittered inter-stimulus interval of 3.0–3.5 s. Stimuli were presented using PsychToolBox (Brainard, 1997; Kleiner et al., 2007). At the start of the flickering stimulus, a trigger code was sent from the stimulus computer to the MEG electronics rack, inserting a trigger into the MEG data that was synced to stimulus onset. A photodiode was used to measure the visual stimulus onset delay, and this delay was accounted for throughout the analysis; thus, all analyses were synced to stimulus onset. Stimuli were back-projected onto a semi-translucent non-ferromagnetic screen positioned 42 in (1.07 m) away from the participant using a Panasonic PT-D7700U-K model DLP projector with a refresh rate of 60 Hz. Each participant completed at least 100 trials, and the task lasted approximately 6 minutes.

### 2.5 MEG data collection

Neuromagnetic data were sampled continuously at 1 kHz using a 306-channel MEGIN system with an acquisition bandwidth of 0.1–330 Hz (Helsinki, Finland). All recordings were conducted in a one-layer magnetically shielded room with active shielding engaged. Prior to MEG measurement, four continuous head position indicator coils were attached to the participant’s head. The locations of these coils, three fiducial points (i.e., the nasion, left and right periauricular), and multiple points capturing the scalp surface were localized using a 3-D digitizer (Fastrak, Polhemus Navigator Sciences, Colchester, VT, USA). Once the participant was positioned for MEG recording, an electric current with a unique frequency label (e.g., 322 Hz) was fed to each of the head position indicator coils. This induced a measurable magnetic field that allowed each coil to be localized relative to the sensors throughout the recording session, which by proxy allowed head position to be continuously monitored relative to the sensor array. After the recording, MEG data from each participant were individually corrected for head motion and subjected to noise reduction using the signal space separation method with a temporal extension (tSSS; Taulu & Simola, 2006; Taulu et al., 2005). Head motion correction was such that the position of the head throughout the recording was aligned to the individual’s head position at the start of the recording.

### 2.6 MRI data collection, MRI processing, and MEG coregistration

Individual structural MRI data were obtained with a 3T Siemens Prisma MRI scanner using a 32-channel head coil with the following parameters: TR, 2400 ms; TE, 1.96 ms; field of view, 256 mm; matrix, 256 × 256; slice thickness, 1 mm; voxel size, 1.0 x 1.0 x 1.0 mm; acquisition plane, sagittal; flip angle, 8 degrees. Structural MRI data were aligned parallel to the anterior and posterior commissures and transformed into the Talairach coordinate system (Talairach & Tournoux, 1988). Because head position indicator coil locations were known in head coordinates, all MEG measurements were able to be transformed into a common coordinate system. Each participant’s MEG data were coregistered with structural T1-weighted MRI data using this common coordinate system prior to source space analyses using BESA MRI (Version 3.0). Following source analysis, each participant’s functional images were transformed into standardized space using the transform applied to the structural MRI volume.

### 2.7 MEG preprocessing

Cardiac, eye blink, and eye movement artifacts were removed from the data using signal-space projection (SSP), which was accounted for during source reconstruction (Uusitalo & Ilmoniemi, 1997). Data were high-pass filtered at 0.3 Hz, and the continuous magnetic time series was then divided into epochs of 2.9 s duration (stimulus onset = 0.0 s), with the baseline being defined as -0.9 to -0.1 s before stimulus onset. Epochs containing artifacts were rejected based on a fixed threshold method per individual, supplemented with visual inspection. Briefly, we used individually determined thresholds based on the signal distribution for both signal amplitude and gradient (i.e., change in amplitude as a function of time) to reject artifacts, which accounts for variability in the distance between the head and the MEG sensor array, as well as differences in actual neural response amplitude, between participants. The average amplitude threshold was 1493.13 (SD = 419.515) fT/cm and the average gradient threshold was 505.13 (SD = 257.551) fT/(cm∗ms). Across the sample, an average of 88.13 (SD = 6.157) trials per participant (out of 100 possible trials) were used for further analysis. There were no significant group differences in number of trials used for analysis, *t*(38) = 1.525, *p* = .135.

### 2.8 MEG sensor-level analysis

Artifact-free epochs were transformed into the time–frequency domain using complex demodulation (resolution: 1.0 Hz, 50 ms; Papp & Ktonas, 1977) from 2 to 100 Hz, and the resulting spectral power estimations per sensor were averaged over trials to generate time–frequency plots of mean spectral density. These sensor-level data were normalized by dividing the power value of each time–frequency bin by the respective bin’s baseline power, calculated as the mean power during the -0.9 to -0.1 s time period. This normalization allowed task-related power fluctuations to be visualized in sensor space.

The time–frequency windows subjected to beamforming (i.e., imaging) in this study were derived through a two-stage statistical analysis of the sensor-level spectrograms across the entire array of gradiometers (magnetometer data were not analyzed), in line with our previous studies (Heinrichs-Graham, Walker, Eastman, et al., 2022; Heinrichs-Graham, Walker, Taylor, et al., 2022; Heinrichs-Graham & Wilson, 2012; Heinrichs-Graham et al., 2017, 2021, 2023). Each data point in the spectrogram was initially evaluated using a mass univariate approach based on the general linear model. To reduce the risk of false positive results while maintaining reasonable sensitivity, a two-stage procedure was followed. In the first stage, one-sample *t*-tests were conducted on each data point and the output spectrogram of *t*-values was thresholded at *p* < .05 to define the time–frequency bins containing potentially significant oscillatory deviations across all participants. In stage 2, time–frequency bins that survived this initial threshold were clustered with temporally and/or spectrally neighboring bins that were also above the (*p* < .05) threshold and found in sensors within 4 cm of each other (i.e., spatial clustering). A cluster value was derived by summing all of the *t*-values of all data points in the cluster. Nonparametric permutation testing was then used to derive a distribution of cluster values, and the significance level of the observed clusters (from stage 1) was then tested directly using this distribution (Ernst, 2004; Maris & Oostenveld, 2007). For each comparison, at least 5000 permutations were computed to build a distribution of cluster values. Based on these analyses, the time–frequency windows that contained significant oscillatory events across all participants were subjected to beamforming.

### 2.9 MEG source-level analysis

Cortical networks were imaged through an extension of the linearly constrained minimum variance (LCMV) vector beamformer (Hillebrand et al., 2005; Liljeström et al., 2005; Van Veen et al., 1997), which employs spatial filters in the frequency domain to calculate source power for the entire brain volume with high spatial accuracy and resolution (Barnes et al., 2004; Hillebrand et al., 2005). In particular, beamforming has been demonstrated to be able to resolve visual oscillatory responses with 5–10 mm spatial resolution (Barnes et al., 2004). Individual images were derived from the cross-spectral densities of all combinations of MEG gradiometers averaged over the time–frequency range of interest and the solution of the forward problem for each location on the grid specified by input voxel space. Following convention, we computed noise-normalized, differential source power per voxel in each participant using active (i.e., task) and passive (i.e., baseline) periods of equal duration and bandwidth (Van Veen et al., 1997). Normalized differential source power was computed for the selected time–frequency bands per participant over the entire brain volume at 4.0 mm^3^ resolution. Each participant’s functional images were then transformed into standardized space using the transform that was previously applied to the structural images. MEG pre-processing and imaging used the Brain Electrical Source Analysis (BESA; version 7.1) software. For the exact time–frequency windows that were imaged, see Results section.

As we were primarily interested in probing entrainment dynamics (i.e., changes in entrainment response power as a function of time), we extracted virtual sensors by applying the sensor weighting matrix derived through the forward computation to the preprocessed signal vector, which yielded two orthogonal time series for each coordinate in source space (Gross et al., 2001). We used the time series with the maximal response for our analyses. Note that this virtual sensor extraction was done per participant, once the coordinates of interest (i.e., peak response to the stimulus) were known. Once the virtual sensor time series were extracted, we computed the envelope of the spectral power in the frequency bin corresponding to the exogenous entrainment frequency (i.e., 15 Hz) and its second harmonic (i.e., 30 Hz) with a time–frequency resolution of 1.0 Hz and 50 ms, and then baseline corrected this time series to yield a relative response time series for each participant.

### 2.10 Statistical analysis

Once relative response time series were created, we then extracted the relative power in each time window during entrainment (i.e., 0.3 to 1.0 s) in 50 ms bins at the entrainment frequency (15 Hz) and the second harmonic (i.e., 30 Hz) and submitted these data to separate linear mixed effects (LME) models, with hearing group as a between-subjects factor and time as a within-subjects factor. We chose this analysis because it allowed us to determine differences in entrainment power (i.e., main effect of group), as well as differences in the slope of entrainment between groups (i.e., time-by-group interaction). We then ran correlations between the visual entrainment characteristics (i.e., power, slope) of both 15 and 30 Hz responses and neuropsychological measures in each group.

In addition, we performed an exploratory whole-brain analysis to investigate the impact of auditory experience on network-level neural dynamics. To this end, we ran whole-brain partial correlations between hearing aid use and source images of the 15 and 30 Hz responses individually, controlling for degree of hearing loss (i.e., BEPTA). All output statistical maps were then adjusted for multiple comparisons using a spatial extent threshold (i.e., cluster restriction; *k* = 5 contiguous 4 mm^3^ voxels) based on the theory of Gaussian random fields (Poline et al., 1995; Worsley et al., 1996, 1999). Basically, statistical maps were initially thresholded at *p* < .001, and then a cluster-based correction method was applied such that at least five contiguous voxels must be significant at *p* < .001 in order for a cluster to be considered significant. Finally, we performed correlations between neuropsychological test scores and neural activity from peaks found in these whole-brain analyses in CHH.

## 3 Results

### 3.1 Participants demographics

Two CNH were excluded from statistical analyses as outliers during source analysis. The final sample included 40 participants, consisting of 23 CNH and 17 CHH. A summary of the measures of both groups is provided in Table 1. The two hearing groups were not significantly different on any demographic and neuropsychological measures (see Table 1). The final sample of CHH had an average BEPTA of 44.82 dB (SD = 15.40 dB, range = 24–76 dB HL) and wore their hearing aids for an average of 80.12 hours/week during the school year (SD = 29.17 h, range = 0–112 h). There was a significant correlation between BEPTA and hearing aid use, *r*(15) = .588, *p* = .013, such that CHH who had greater severity of hearing loss also wore their hearing aids more often.

**Table 1.** Summary of participant demographics and neuropsychological assessments.

	CNH		CHH			
	avg. (SD)	range	avg. (SD)	range	Statistic	*p* value
Age (years)	11.42 (2.47)	7.21–15.93	12.14 (2.24)	7.41–15.77	0.954	.346
Sex (M/F)	15 M/8 F	-	11 M/6 F	-	0.001	.973
Handedness (R/L/Am)	21 R/2 L	-	13 R/3 L/1 Am	-	1.687	.194
WASI-II VCI SS	109.04 (13.04)	81–138	103.35 (9.54)	90–124	-1.521	.136
WASI-II PRI SS	105.57 (12.83)	71–135	107.94 (11.09)	87–130	0.612	.544
WASI-II FSIQ-4 SS	108.43 (11.04)	77–133	106.47 (9.59)	91–123	-0.588	.560
D-KEFS LF SS	9.43 (3.53)^a^	4–19	9.73 (2.71)^a^	5–15	0.280	.781
D-KEFS CF SS	11.00 (3.81)^a^	3–19	11.00 (3.30)^a^	5–19	0.000	1.000
ADHD AP raw	3.36 (0.83)^a^	1–4.75	3.65 (0.79)^b^	2.25–5	0.426	.672
ADHD CB raw	3.86 (1.02)^a^	1–5	3.70 (0.80)^b^	2.5–5	-0.501	.620
BEPTA (dB)	-	-	44.82 (15.40)	24–76	-	-
Hearing aid use (hours/week)	-	-	80.12 (29.17)	0–112	-	-
Maternal education (years)	16.09 (2.16)^c^	12–20	15.59 (2.09)	12–20	-0.731	.470

a Missing two participants’ data, n = 21.

b Missing one participant’s data, n = 16.

c Missing one participant’s data, n = 22.

Abbreviations are as follows: Sex: M = male, F = female; Handedness: R = right-handed, L = left-handed, Am = ambidextrous; WASI-II = Wechsler Abbreviated Scale of Intelligence, VCI SS = verbal comprehension index standard score, PRI = perceptual reasoning index standard score, FSIQ-4 = full-scale intelligence quotient standard score; D-KEFS = Delis–Kaplan Executive Function System test, LF raw = letter fluency raw score, CF = category fluency; ADHD = Vanderbilt ADHD Scale Questionnaire, AP = academic performance, CB = classroom behavior; BEPTA = better-ear pure tone average.

### 3.2 Sensor- and source-level results

**Fig. 1.**
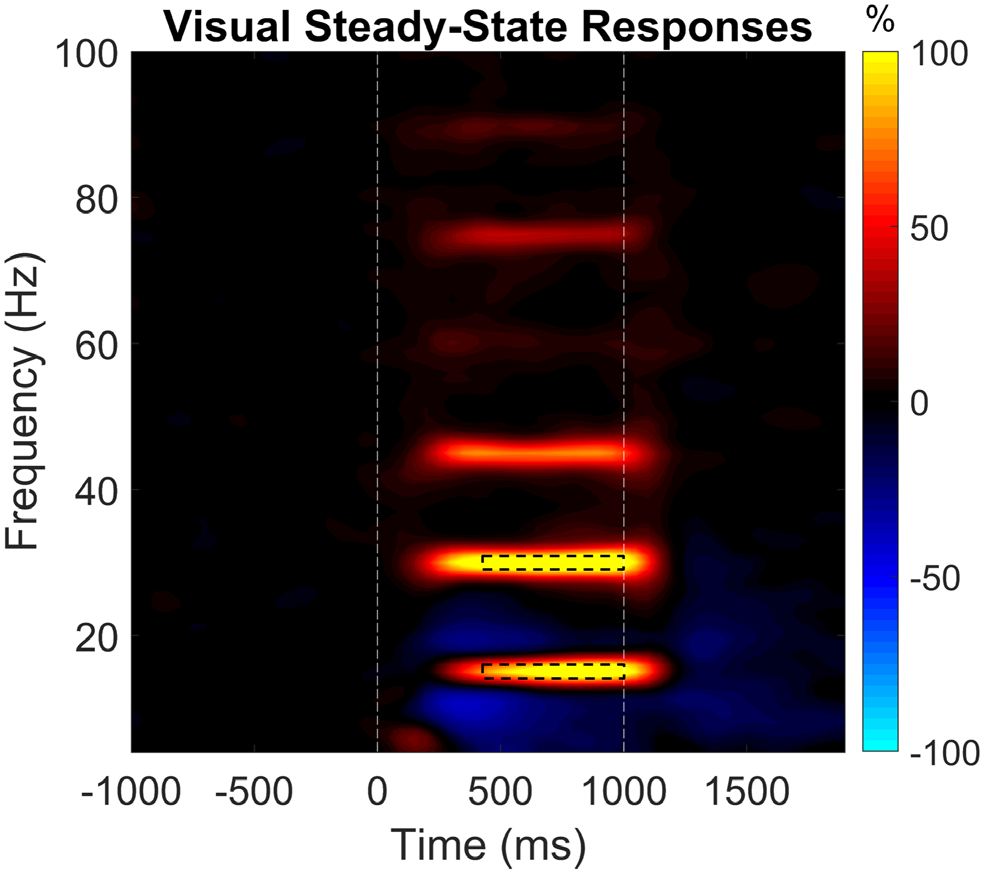
Time–frequency spectrogram from a sensor near the occipital cortex, showing neural responses to 15-Hz flicker stimulation. White dotted lines demarcate the onset (0.0 s) and offset (1.0 s) of the flickering dot. Black dotted boxes show the beamforming windows, 14–16 and 29–31 Hz by 0.3–1.0 s.

**Fig. 2.**
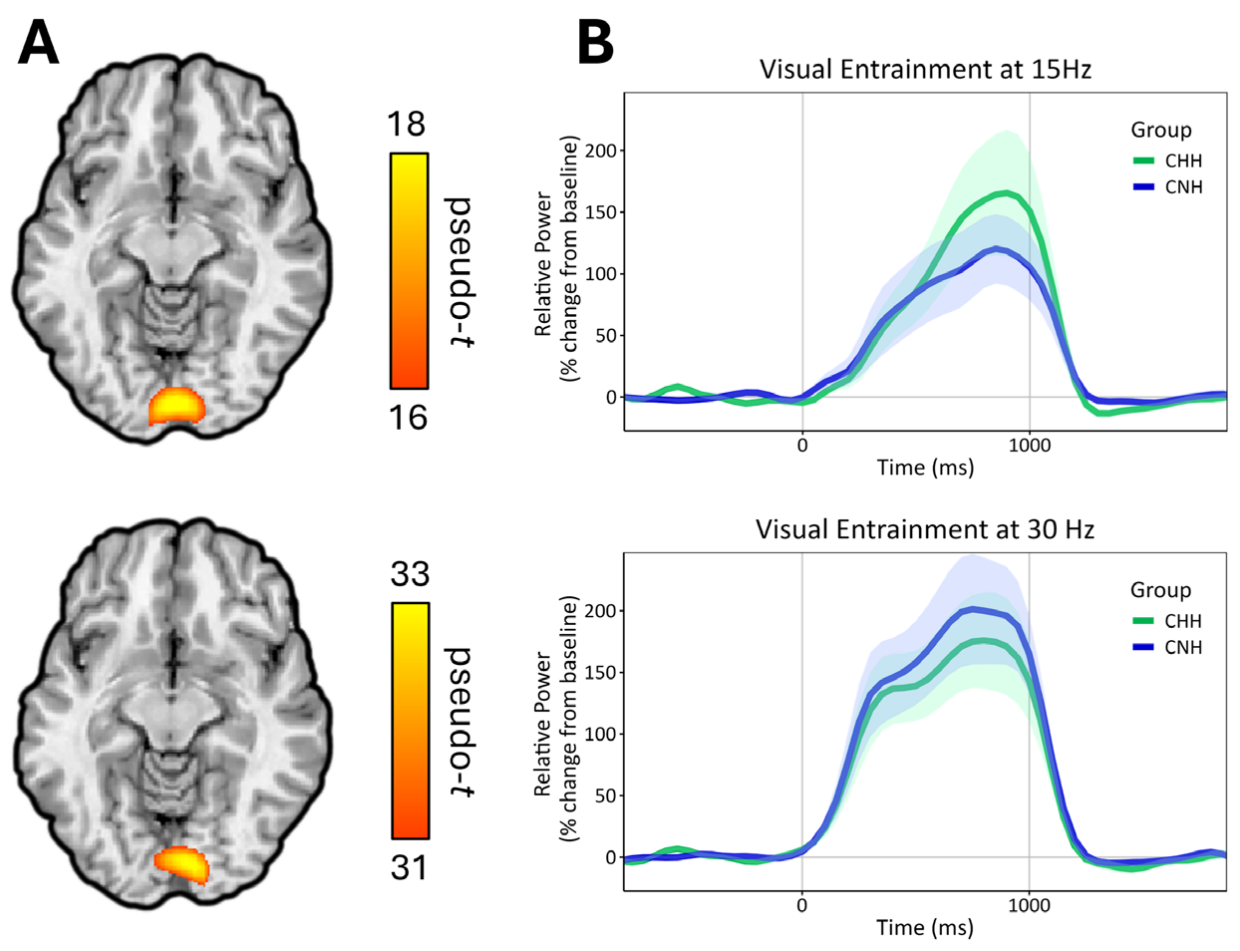
(A) Grand-averaged beamformer images showing peak activity in the primary visual cortex at the fundamental frequency (15 Hz) (top) and the second harmonic (30 Hz) (bottom). (B) Virtual sensor time series from the peak region at 15 Hz (top) and 30 Hz (bottom) for CHH (green) and CNH (blue).

Statistical analysis of sensor-level time–frequency data revealed significant increases in neural activity at the stimulation frequency (15 Hz) and its harmonics (30 Hz, 45 Hz, etc.) from shortly after the stimulus onset to approximately 0.2 s after stimulus offset (*p* < .001; Fig. 1). Of note, we were unable to resolve a significant response at 60 Hz because this is the frequency of power mains in the United States. Next, we imaged the peak time window of significant responses to the stimulation frequency (14–16 Hz, 0.3–1.0 s) and the second harmonic (29–31 Hz, 0.3–1.0 s), and then grand-averaged each response across participants to find regions of peak activity, which were centered in the medial occipital cortex near the calcarine fissure for both the 15 and 30 Hz responses (Fig. 2A). As described above, we extracted the relative spectral envelope for 14–16 and 29–31 Hz separately for the duration of entrainment (0.3–1.0 s, stimulus onset = 0.0 s) in 50-ms time bins to obtain the relative response power as a function of time for each participant (Fig. 2B).

### 3.3 Effects of hearing status on 15 and 30 Hz entrainment responses

The extracted time series from the peak voxels for both 15 and 30 Hz entrainment were each submitted to linear mixed effects modeling. As described above, the power in 50-ms bins from 0.3 to 1.0 s was extracted and these data were submitted to a linear mixed effects model with time (14 bins total) as a within-subjects factor and group as a between-subjects factor. Of note, hemisphere was not considered, as the peak voxel for each response was along the midline. For the 15 Hz entrainment response, we found a significant group-by-time interaction (β = -1.055 × 10^-3^, SE = 2.355 × 10^-4^, *p* < .001), such that increases in entrainment power as a function of time were greater for CHH than for CNH, suggesting altered entrainment dynamics between groups (Fig. 2). There was also a significant main effect of time (β = 2.099 × 10^-3^, SE = 1.786 × 10^-4^, *p* < .001), indicating the entrainment power response increased as a function of time, but no significant main effect of group (β = 4.400 × 10^-1^, SE = 4.499 × 10^-1^, *p* = .333). For the 30 Hz entrainment response, we found a significant main effect of time (β = 8.170 × 10^-4^, SE = 1.976 × 10^-4^, *p* < .001). However, there was no significant main effect of group (β = 4.390 × 10^-2^, SE = 5.253 × 10^-1^, *p* = .934), nor was there a significant group-by-time interaction (β = 2.654 × 10^-4^, SE = 2.605 × 10^-4^, *p* = .309). The statistics for both models are summarized in Table 2.

**Table 2.** Statistics summary of the linear mixed effects model of the neural response power of the 15 and 30 Hz ERS as a function of time (i.e., slope) and group.

	Parameter	Coefficient Estimate (β)	Standard Error	*p* value
15 Hz	*Intercept*	-1.560 × 10^-1^	3.412 × 10^-1^	.650
	*Group*	4.400 × 10^-1^	4.499 × 10^-1^	.333
	***Time*** ^***^	**2.099** **×** **10^-3^**	**1.786** **×** **10^-4^**	**2** **×** **10^-16^**
	***Group*** ***×*** Time^***^	**-1.055** **×** **10^-3^**	**2.355** **×** **10^-4^**	**9.25** **×** **10^-6^**
30 Hz	*Intercept* ^*^	1.028	3.983 × 10^-1^	**.013**
	*Group*	4.390 × 10^-2^	5.253 × 10^-1^	.934
	***Time*** ^***^	**8.170** **×** **10^-4^**	**1.976** **×** **10^-4^**	**4.13** **×** **10^-5^**
	*Group* *×* *Time*	2.654 × 10^-4^	2.605 × 10^-4^	.309

Significant effects are shown in bold. ***p < .001, *p < .05.

### 3.4 Impact of auditory experience on cross-modal neural activity

**Fig. 3.**
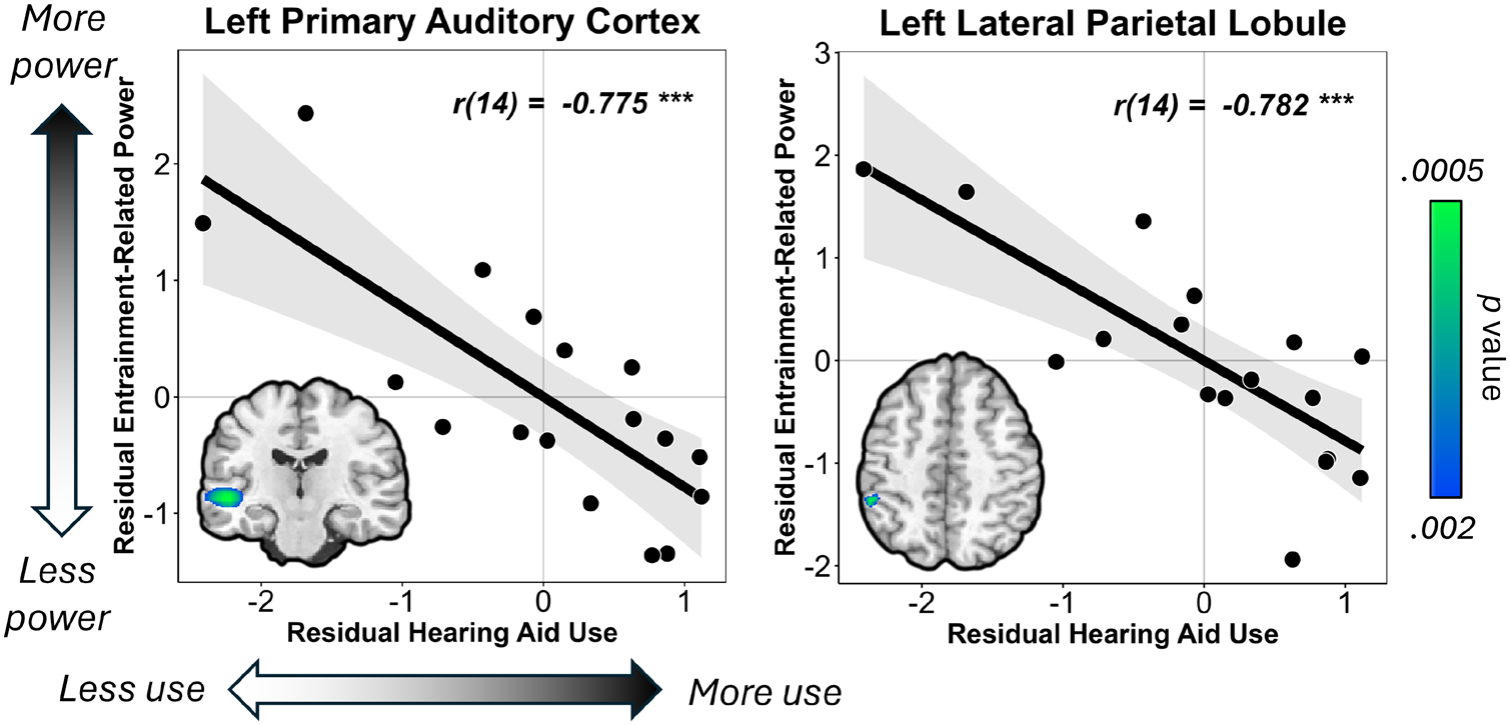
Whole-brain correlations between entrainment power and hearing aid use, controlling for degree of hearing loss. Residuals of response power are shown on the y-axis, while residuals of hearing aid use are found on the x-axis. Whole-brain statistical maps showing peak differences are inset on each plot, with color bars denoting significance. Images are thresholded at *p* < .002 to aid in visualization; however, both peaks survived at *p* < .001, corrected.

Given the literature demonstrating cross-modal neuroplasticity in the auditory cortices of individuals with severe-to-profound hearing loss during visual processing (Kral & Sharma, 2023; Sharma & Glick, 2016; Sharma et al., 2015), we probed the impact of auditory experience on whole-brain dynamics during visual entrainment processing in CHH. To this end, we ran whole-brain partial correlations between 15 Hz activity and the average hours of weekly hearing aid use, controlling for degree of hearing loss. We found that weekly hearing aid use was significantly correlated with the entrainment-related neural activity in the left transverse temporal gyrus (i.e., primary auditory cortex; *r*(14) = -.775, *p* < .001, corrected), and left lateral parietal cortex (*r*(14) = -.782, *p* < .001, corrected; Fig. 3), after controlling for degree of hearing loss. In these regions, CHH who wore their hearing aids less often recruited these non-visual (e.g., auditory, multisensory) areas during visual entrainment, while those who wore their hearing aids more often did not. Of note, there was a significant correlation between degree of hearing loss and 15 Hz entrainment-related activity in the left lateral parietal lobule (*p* > .001); however, this area was still significantly related to hearing aid use after covarying out degree of hearing loss, suggesting that auditory experience was uniquely related to cross-modal neuroplasticity above and beyond any effects of degree of hearing loss. There were no significant correlations between hearing aid use and 30 Hz entrainment activity after controlling for degree of hearing loss.

### 3.5 Cross-modal neural activity and behavior in CHH

**Fig. 4.**
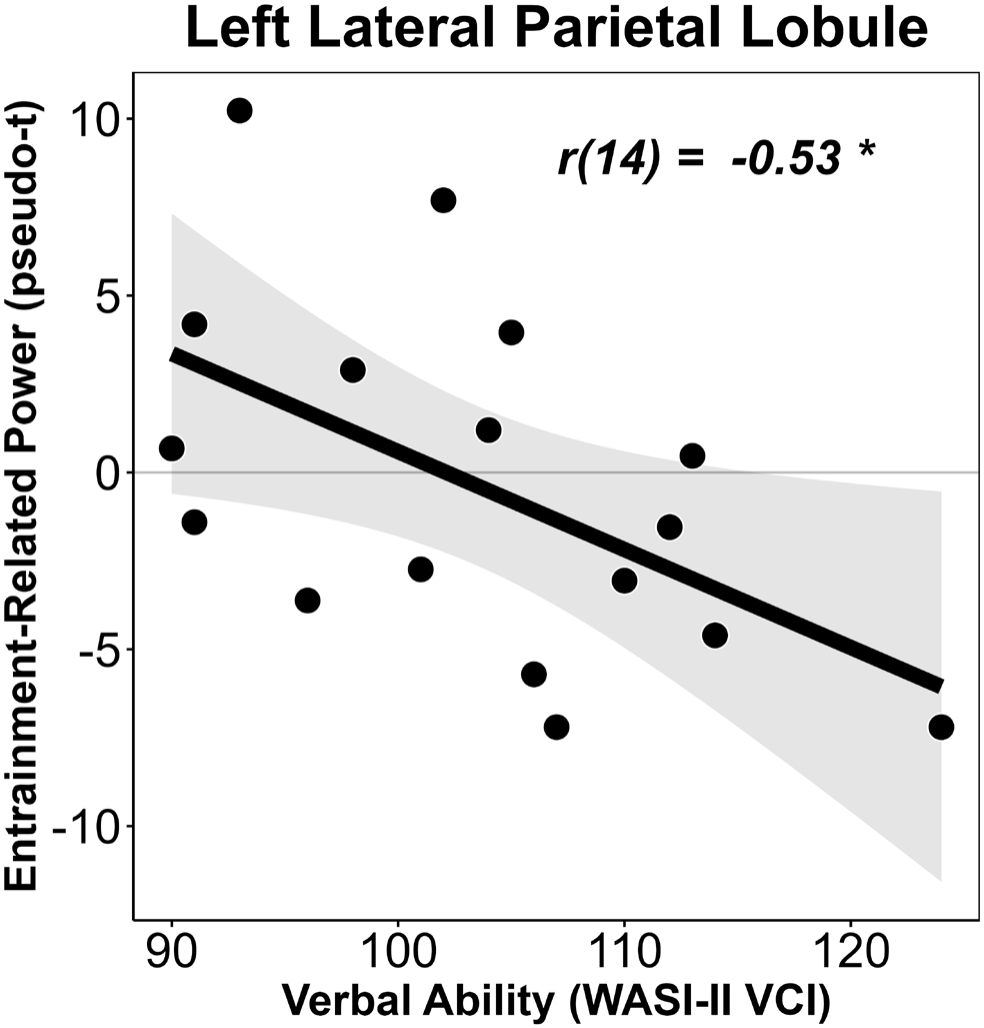
Correlation between lateral parietal entrainment-related activity and verbal ability in CHH. Neural activity is shown on the y-axis, while VCI score is shown on the x-axis. *p < .05.

Finally, we sought to determine whether cross-modal neural activity was related to behavioral outcomes in CHH. To this end, we ran correlations between neuropsychological test scores and peaks of neural activity in the left primary auditory cortex and lateral parietal cortex extracted from the whole-brain partial correlation analyses described above. We found a significant correlation between left lateral parietal entrainment-related activity and the Verbal Composite Index (VCI) on the WASI-II, *r*(16) = -.530, *p* = .029 (Fig. 4). There were no other significant correlations between cross-modal entrainment-related activity and neuropsychological test performance in CHH.

## 4 Discussion

In this study, we probed the impact of mild-to-severe hearing loss on the neural dynamics underlying visual entrainment in youth. We found that CHH exhibited altered 15 Hz entrainment dynamics in the primary visual cortex compared with CNH; while both groups showed a positive entrainment slope, response power continued to increase as a function of time to a greater extent in CHH than in CNH, suggesting a lack of entrainment stability. Perhaps most interestingly, our follow-up whole-brain analysis showed a significant relationship between average hours of hearing aid use and activity in the left primary auditory cortex and left lateral parietal cortex in CHH, such that those who wore their hearing aids the least recruited these cross-modal regions the most, whereas those who wore their hearing aids the most did not. Finally, decreased power in the lateral parietal cortex was significantly correlated to better verbal ability in CHH. Below we discuss the implications of these findings in our understanding of how differences in auditory experience impact the brain dynamics serving visual processing and how these alterations may relate to cognitive and language outcomes in CHH.

We first sought to determine whether CHH showed altered visual entrainment in the primary visual cortices relative to CNH using linear mixed modeling. While both groups showed a significant linear increase in 15 Hz power in the primary visual cortices in response to the entrainment stimuli, we found a significant group-by-time interaction, such that CHH showed a steeper increase in response power as a function of time than CNH. This pattern of results suggests a decreased stability of the entrainment response in CHH; in other words, entrainment power became more stable (i.e., less change as a function of time) throughout the entrainment period in CNH, while entrainment power continued to increase in CHH. This pattern could be visualized in the averaged time series. To our knowledge, our study is the first to look at the changes in neural entrainment response as a function of time in individuals with hearing loss (in contrast to investigations of overall response power across time, e.g., Stroh et al., 2022), and thus it is difficult to directly compare with the literature in this domain. However, from a neurobehavioral standpoint, previous studies have found that individuals with severe-to-profound hearing loss exhibit a myriad of enhancements in visual processing ability (Alencar et al., 2019; Bottari et al., 2010; Dye, 2014; Tharpe et al., 2008), and it is likely that altered neural responsivity to visual input underlies these enhancements. To date, it is unclear whether alterations in visual processing ability extend into CHH, as there is a paucity of literature investigating the effects of mild-to-severe hearing loss on visual processing (Bell et al., 2020; Jerger et al., 2020; Lalonde & McCreery, 2020). Nonetheless, we hypothesize that the continued increase in occipital power in response to a constant, repetitive stimulus in CHH found in this study may be reflective of reduced visual habituation, which may be compensatory in order to enhance detection and selection of visual input. Reduced habituation to visual input could be functionally beneficial in CHH, who experience lower fidelity auditory input relative to their CNH peers and thus must reliably and consistently detect and process sensory input in other domains in order to maximize their perception of our multisensory world. Indeed, a recent comprehensive review underscores the theoretical importance of neuronal entrainment mechanisms in selecting, prioritizing, and modulating sensory input and suggests that the brain’s ability to entrain to an external stimulus is crucial for goal-directed cognitive actions (Lakatos et al., 2019). We extend this theory in the context of hearing loss and suggest that alterations in visual entrainment dynamics in CHH could serve as a compensatory mechanism by which CHH can better sense and process their environment. Of course, these interpretations are largely speculative given the limited scope of our study, and larger investigations of the associations between sensory entrainment and cognitive ability in CHH are necessary to determine how entrainment dynamics could predict behavior in this population.

Crucially, we also found a significant partial correlation between hearing aid use and entrainment-related activity in the left primary auditory cortex, controlling for degree of hearing loss. These data are the first to show that auditory experience is related to the amount of cross-modal neuroplasticity in CHH and suggest that those who wear their hearing aids the least (i.e., have less consistent auditory experience) show greater recruitment of auditory-related regions in response to a visual stimulus. However, those who wear their hearing aids the most show little-to-no recruitment of these regions during visual entrainment, suggesting a normalization of sensory-related activity. In deaf individuals who have severely reduced or absent auditory input, there are neuroplastic changes such that other sensory domains (e.g., visual, somatosensory) become represented in the primary auditory cortices (Alencar et al., 2019; Bottari et al., 2010; Gordon et al., 2011; Sharma et al., 2015). Some studies link this increase in cross-modal neuroplasticity to improvements in visual processing outcomes (Alencar et al., 2019; Bednaya et al., 2021; Retter et al., 2019). However, other work demonstrates that improved access to sound following cochlear implantation leads to a decrease in the representation of visual stimuli in the primary auditory cortices (Kral & Sharma, 2023; Sharma & Glick, 2016; Sharma et al., 2015), and the amount of decrease in cross-modal neural representation is related to improved speech outcomes (Doucet et al., 2006; Paul et al., 2024). Thus, there seems to be a dynamic, time-sensitive neuroplastic mechanism by which the brain responds to differences in sensory input in individuals who are deaf. Nonetheless, it is important to note that while age of implantation can vary across individuals, cochlear implantation is often interpreted in the literature as largely an all-or-nothing intervention, such that once an individual receives a cochlear implant, it is assumed that they have an immediate change in their auditory access moving forward. In contrast, there has recently been a heightened focus on the individual variability in auditory access in youth who wear hearing aids, due in part to the substantial variability in both hearing aid use and aided audibility (i.e., how well hearing aids restore access to speech sounds) (Moeller & Tomblin, 2015). One large-scale study found that approximately 25% of school-aged CHH do not wear their hearing aids at least 8 h per day (Walker et al., 2013; Walker, McCreery, et al., 2015). The variability in hearing aid use in CHH provides a unique opportunity to investigate how individual differences in the amount of auditory input relate to neural activity and behavioral outcomes, and indeed, our data support a linear relationship between the amount of auditory experience and the amount of cross-modal activity in the auditory cortices during visual processing in CHH. In sum, our results suggest an increase in cross-modal plasticity in the primary auditory cortices in CHH who do not wear their hearing aids full-time that is reduced or eliminated in CHH who use their hearing aids consistently. These data complement and expand the current literature on cross-modal neuroplasticity (Bauer et al., 2020; Kujala et al., 2000; Sharma & Glick, 2016) to include youth with milder forms of hearing loss. Future studies should investigate the developmental trajectories of these patterns in CHH and determine what the neurobehavioral consequences of these relationships are.

Interestingly, we also found a significant correlation between hearing aid use and entrainment-related activity in the left lateral posterior parietal cortex, and activity in this region also significantly correlated with verbal ability in CHH. The lateral posterior parietal cortex has been reliably associated with multimodal processing (Sereno & Huang, 2014; Ursino et al., 2014), including visuotactile (Limanowski & Blankenburg, 2018) and audiovisual integration (Bolognini et al., 2009; Regenbogen et al., 2018), as well as a myriad of other cognitive functions including visuospatial representation and attention (Bruner et al., 2023; Corbetta & Shulman, 2011; Kemmerer et al., 2022), speech processing (Hickok & Poeppel, 2004; Poeppel, 2014), and semantic and visual memory (Humphreys et al., 2022) processes. Thus, it is possible that activity in the left lateral posterior parietal cortex served to integrate information between the visual and auditory cortices during the task, especially in those CHH who wear their hearing aids the least and thus show the greatest entrainment-related cross-modal activity in the auditory cortices. In other words, stimulus-related entrainment information may be transferred and integrated between the primary sensory cortex (i.e., visual cortex) and the cross-modal sensory cortex (i.e., temporal cortex) via multisensory integration-related regions of the lateral parietal cortices (Bauer et al., 2020). Interestingly, this cross-modal integration-related activity was negatively related to verbal outcomes in CHH, such that those who showed the least cross-modal activity in this region also showed the highest verbal scores. Taken together, our data suggest that increased auditory access via hearing aids is not only associated with a decrease in cross-modal activity, but also the degree to which this cross-modal activity is suppressed in these areas is related to better verbal outcomes. Nonetheless, these data are correlational in nature, and thus, our interpretations must be taken largely as speculative. Future studies should more directly investigate the causal relationships between auditory access, cross-modal neuroplasticity, and behavioral outcomes in CHH. Moreover, clarification of how lateral parietal activity supports visual processing and audiovisual integration in CHH would benefit from structural and functional connectivity analyses; this should be prioritized as a future direction.

In sum, the current study provides compelling evidence of altered visual entrainment processing in CHH compared with CNH that is sensitive to individual differences in auditory experience. We found altered visual entrainment dynamics in the primary visual cortices, such that CHH showed a significantly larger increase in entrainment-related power as a function of time during stimulus presentation. Moreover, we found significant correlations between hearing aid use and cross-modal response power in both the primary auditory cortex and lateral parietal cortex, such that CHH who wore their hearing aids the least showed the greatest degree of cross-modal neuroplasticity, which was largely absent in CHH who wore their hearing aids consistently. These data provide important new information regarding the impact of hearing loss and hearing aid intervention on visual processing and neural development in CHH, and may eventually hold promise as a passive, quantitative neural marker by which improvements in hearing intervention technology and therapeutic approaches can be assessed.

## Data and Code Availability

The data that support the findings of this study are stored on the COllaborative Informatics and Neuroimaging Suite (https://coins.trendscenter.org/) and are available from the corresponding author on reasonable request.
